# Mortality after mechanical thrombectomy in anterior circulation stroke may be higher at nighttime and on weekends

**DOI:** 10.1007/s00330-020-07615-w

**Published:** 2020-12-19

**Authors:** C. Zaeske, L. Goertz, J. Kottlors, M. Schlamann, O. A. Onur, V. Maus, A. Mpotsaris, T. Liebig, R. Forbrig, C. Kabbasch, N. Abdullayev

**Affiliations:** 1 Institute for Diagnostic and Interventional Radiology, Faculty of Medicine, University Hospital Cologne, Kerpener Strasse 62, 50937 Cologne, Germany; 2 Center for Neurosurgery, Faculty of Medicine, University Hospital Cologne, Cologne, Germany; 3 Department of Neurology, Faculty of Medicine, University Hospital Cologne, Cologne, Germany; 4 Institute of Diagnostic and Interventional Radiology, Neuroradiology and Nuclear Medicine, Knappschaftskrankenhaus Bochum, Ruhr University Bochum, Bochum, Germany; 5 Department of Neuroradiology, University Hospital RWTH, Aachen, Germany; 6 Department of Neuroradiology, University Hospital Munich (LMU), Munich, Germany

**Keywords:** Radiation exposure, Radiology interventional, Thrombectomy

## Abstract

**Objectives:**

The objective of this study was to compare clinical outcome and procedural differences of mechanical thrombectomy (MT) during on-call with regular operating hours. We particularly focused on dosimetric data which may serve as potential surrogates for patient outcome.

**Methods:**

A total of 246 consecutive patients who underwent MT in acute anterior circulation stroke between November 2017 and March 2020 were retrospectively included. Patients treated (1) during standard operational hours (*n* = 102), (2) daytime on-call duty (*n* = 38) and (3) nighttime on-call duty (n = 106) were compared with respect to their pre-interventional status, procedural specifics, including dosimetrics (dose area product (DAP), fluoroscopy time and procedural time), and outcome.

**Results:**

The collectives treated outside the regular operational hours showed an increased in-hospital mortality (standard operational hours 7% (7/102), daytime on-call duty 16% (6/38), nighttime on-call duty 20% (21/106), *p* = 0.02). Neither the dosimetric parameters nor baseline characteristics other procedural specifics and outcome parameters differed significantly between groups (*p* > 0.05 each). In most cases (> 90%), a successful reperfusion was achieved (TICI ≥ 2b).

**Conclusions:**

We found an increased in-hospital mortality in patients admitted at night and during weekends which was not explained by technical aspects of MT.

**Key Points:**

*• There is an increased mortality of stroke patients admitted at night and on weekends.*

*• This is not explained by technical aspects of mechanical thrombectomy.*

*• There were no statistical differences in the comparison of parameters linked to the radiation exposure, such as DAP, fluoroscopy time and procedure time.*

## Introduction

Previous studies suggested that hospital admissions and/or procedures performed at night and on weekends may be associated with worse patient outcome when compared to admissions during routine daytime shift. This was subsequently described as the “weekend effect” [1]. Despite some controversy on the subject, the effect was demonstrated even after statistical consideration of the severity of the respective disease [1]. Initial data in the field of neuroradiology also show a demonstrable effect on the treatment of patients with acute ischemic stroke (AIS): Mpotsaris et al reported a significant increase of the door-to-reperfusion time in patients admitted at night or weekend [2]. Furthermore, Ogbu et al found an increased mortality rate in the same collective [3].

The cause of the effect has not yet been determined with absolute certainty. Possible reasons are, for example, a reduction of both the medical staff and availability of resources and organising factors outside routine working times [1]. Moreover, due to the human circadian rhythm, cognitive performance varies throughout the day and usually reaches its lowest point at night, possibly yielding reduced overall quality of patient management [3]. In addition to this, it has been shown that a procedure time of more than one hour (known as “golden hour”) is associated with a higher complication rate [4, 5]. Furthermore, radiation exposure per se entails a certain health risk. Guidelines of non-medical radiation safety literature indicate that deterministic effects can occur from a radiation exposure of 15 mSv [6]. However, in the context of neuroradiological interventions, as with all sources of radiation exposure, stochastic effects also occur, with the most frequent and significant risk being cancer [6].

Since mechanical thrombectomy (MT) represents the gold standard in AIS patients with large vessel occlusion (LVO) and is increasingly being implemented in clinical routine worldwide, diagnostic reference levels (DRL) offered by the Federal Office for Radiation Protection serve as a framework for quality control as well as for the radiation dose optimisation of both patients and medical staff. The current national DRL for fluoroscopically guided endovascular thrombus aspiration in AIS patients due to LVO is 180 Gy cm^2^ [7]. Recently, several studies investigated the dosimetric distributions of MT [8–11]. However, the intervention time (routine working daytime vs. on-call duty at night and at the weekend) which may serve as a potential confounder of patient outcome has not been considered in these studies.

In this study, we compared pre-interventional status, procedural specifics (including the dosimetrics dose area product (DAP), fluoroscopy time and procedural time) and outcome during (a) standard working hours on weekdays, (b) daytime on-call duty at weekends/public holidays or (c) nighttime on-call duty. Our objective was to compare clinical outcome and procedural differences of mechanical thrombectomy (MT) during on-call compared to regular operating hours to evaluate if there is a deterioration of clinical outcome during on-call working hours and whether this is reflected in the procedural characteristics of MT.

## Methods

This is a retrospective analysis of 246 consecutive patients who underwent a MT due to LVO in the anterior circulation with consecutive AIS, using an anonymised database. The patients were treated between October 2017 and March 2020. The study was approved by the local ethics committee (Registration ID 16–347) and was conducted in accordance with the Declaration of Helsinki.

### Patient treatment

Inclusion criteria for MT were (1) occlusion of proximal large cerebral arteries of the anterior circulation, proven by computed tomography angiography (CTA) or magnetic resonance angiography (MRA); (2) a baseline National Institutes of Health Stroke Scale (NIHSS) ≥ 5 or aphasia; (3) onset of symptoms within the last 8 h. Exclusion criteria were defined as follows: (1) presence of intracranial haemorrhage (ICH); (2) large completed territorial infarction, confirmed by an Alberta stroke program early CT score (ASPECTS) score of < 5 in the pretherapeutic native cranial computed tomography (NCCT) or by perfusion match (cerebral blood flow versus volume) in CT perfusion; and (3) patients who received concomitant intra-/extracranial stenting and/or percutaneous balloon angioplasty.

The administration of intravenous thrombolysis was independent of MT and performed according to national neurological guidelines

All procedures were performed under general anaesthesia on a biplanar angiography suite (Allura Xper FD 20/15, Philips), using a standardised approach through a transfemoral arterial access, with an 8-French (F) guiding catheter placed in the internal carotid artery. A 6F aspiration catheter was then inserted coaxially, followed by either MT by aspiration alone or in a combined technique with stent-retrieval and aspiration (Solumbra or SAVE technique) [12–14] as a first-line approach. Choice of both material and endovascular technique as well as optimisation of the irradiation field was at the discretion of the treating neurointerventionalist. Stent-retrievers used were the Solitaire stent-retriever (Medtronic), Trevo XP ProVue Retriever (Stryker) and the EmboTrap Revascularization device (Cerenovus) in combination with a Sofia catheter (Microvention).

The interventions were performed by the same amount of medical staff both within and outside regular operational hours, usually consisting of a board-certified interventional radiologist or neuroradiologist and a radiological technician. To investigate the potential impact of the interventionalist’s experience on the irradiation parameters, it was recorded whether the respective interventionalist had less or more than 1 year of experience in neurointerventional procedures.

### Data acquisition

The evaluated data of each patient included demographics and relevant cardiovascular risk factors (arterial hypertension, diabetes, atrial fibrillation) which were assessed by the treating physician and retrospectively obtained from patient charts and medical reports. This also applies for the modified Rankin scale (mRS) score and National Institutes of Health Stroke Scale (NIHSS) score at admission and discharge and mRS after 90 days, including the cause of death, if applicable, which were evaluated by the attending neurologist.

In the reports and images of the pretherapeutic, NCCT and CTA were analysed for diagnosis of the vessel occlusion site, ASPECTS calculation and ICH exclusion. Postinterventional imaging (18 to 24 h after treatment) was analysed with respect to ICH and postinterventional ASPECTS. In case of postinterventional MRI imaging, the ASPECTS was determined using diffusion weight imaging (DWI). Symptomatic intracerebral haemorrhage (sICH) was defined according to the European cooperative acute stroke study (ECASS) III criteria as ICH with deterioration of the NIHSS score by 4 points that was not attributed to other causes [15]. Accordingly, the mRS and NIHSS score were determined at the end of the hospital stay and the mRS score after 90 days.

Recorded procedural data included the number of MT maneuvers, intravenous thrombolysis and the amount of contrast medium used, which was taken from the angiographic reports. These data were analysed by an experienced radiologist (7 years of experience). Degree of reperfusion, assessed with the TICI (thrombolysis in cerebral infarction) [16] score, was taken from the angiographic report and re-graded by a board-certified neuroradiologist (8 years of experience) who was blinded to the clinical outcome.

Fluoroscopy time and dose area product (DAP) were recorded for each procedure by a medical technical radiology assistant at the end of the procedure and retrospectively assessed via the examination protocol. Fluoroscopy time included the time from both detectors of biplane angiographic systems in minutes. DAP comprised the radiation exposure caused by digital subtractions angiography (DSA), roadmap function and DSA roadmap. All images were acquired with a low-dose protocol using Clarity IQ dose reduction software (Philips).

With regard to time intervals, periods from onset of symptoms to groin puncture, from admission to groin puncture, from admission to reperfusion and from groin puncture to reperfusion (procedural time) were examined. Admission time was defined as the point of time, when the patient data was entered into the electronic data management system at arrival in the emergency room. Angiographic and procedural data such as time of groin puncture and time of reperfusion were either given in the angiographic reports or obtainable from automatically created time seals (acquisition time) on DSA images. In patients where the exact time of onset of symptoms could not be determined within the last 8 h, the time interval “onset of symptoms to groin puncture” could not be determined. This is indicated accordingly in the Results section.

### Time of treatment

The included patients were divided into three collectives: (1) Patients who were treated during standard operational hours (Monday to Friday, 08:00–17:59), (2) patients who were treated during daytime on-call duty periods on weekends and public holidays (Saturday and Sunday, 08:00–17:59) and (3) patients who were treated during nighttime on-call duty periods (18:00–07:59). Interventions that started and ended between 08:00 and 17:59 were assigned to the standard operational hours or daytime on-call duty collective, depending on the weekday. As soon as an intervention started during or extended into the night period, it was assigned to the nighttime on-call duty collective.

### Statistical analysis

Normal distribution was tested using the Shapiro-Wilk test. A two-sided *p* value of < 0.05 was considered as statistically significant. Differences in categorical data were identified using Pearson’s chi^2^ or Fisher exact test.

Variances among and between the three categorical variables concerning the continuous data were compared using an ANOVA for parametric data. Variance homogeneity of the data was analysed by the Brown-Forsythe test. Subsequently, post hoc testing for multiple comparisons was done by Dunnett T3 test. On the other hand, Kruskal-Wallis test was applied for the non-parametric data using post hoc Dunn’s test to correct for multiple comparisons.

Additionally, multiple linear regression analyses for the parameters radiation exposure, fluoroscopy time and procedural time were performed. Regarding the mortality, a multiple logistic regression analysis was conducted.

Parametric results are presented as means including standard deviation. Scores and time intervals are presented as median and corresponding interquartile range (IQR). Statistical analysis was performed using GraphPad Prism version 8.0.0 for Windows (GraphPad Software).

## Results

### Demographics and stroke characteristics

Out of a total of 246 patients, 102 (41%) were treated during standard operational hours, 38 (15%) during daytime on-call duty and 106 (43%) during nighttime on-call duty. There were no significant differences regarding age, gender or cardiovascular risk factors as detailed in Table 1 (*p* > 0.05 each). Also, with regard to stroke-related characteristics, no significant differences were found concerning localisation of the occlusion site, the scores obtained on admission (ASPECTS, mRS, NIHSS) or the time interval between onset of symptoms and admission (*p* > 0.055; Table 1). The exact onset could not be determined in two cases of the standard operational hours collective.

**Table 1:** Baseline characteristics

	Standard working hours (*n* = 102)	On-call duty, daytime (*n* = 38)	On-call duty, nighttime (*n* = 106)	
Age (years)	75.29 ± 14.04	74.45 ± 12.73	75.40 ± 12.98	*p* = 0.81
Gender				
Female	48 (47%)	21 (55%)	51 (48%)	*p* = 0.68
Male	54 (53%)	17 (45%)	55 (52%)	
Occlusion site				
M1	83 (81%)	32 (84%)	84 (79%)	*p* = 0.79
M2	19 (18%)	6 (16%)	22 (21%)	
Scores at admission				
ASPECTS	9 (8–10)	10 (8–10)	9 (8–10)	*p* = 0.98
mRS	5 (4–5)	5 (4–5)	5 (4–5)	*p* = 0.90
NIHSS	15 (12–21.75)	15 (12–21)	17 (12–21.75)	i = 0.42
Interval onset to admission (min) (exclusion of 2 patients, *n* = 244)	112.8 ± 84.44 (*n* = 100)	142.2 ± 123.7	117.3 ± 76.12	*p* = 0.49
Cardiovascular risk factors				
Arterial hypertension	63 (62%)	24 (65%)	69 (65%)	*p* = 0.87
Atrial fibrillation	58 (57%)	26 (70%)	64 (60%)	*p* = 0.36
Diabetes	18 (18%)	7 (19%)	21 (20%)	*p* = 0.92

*M1*, M1-segment of middle cerebral artery; *M2*, M2-segment of middle cerebral artery

### Procedural details and timings

Procedural details are shown in Table 2. There was a tendency towards a higher rate of MT manoeuvres at night, which did not reach statistical significance (*p* > 0.05). There were no significant differences regarding the median DAP (*p* > 0.05).

**Table 2:** Procedural details

	Standard working hours (*n* = 102)	On-call duty, daytime (*n* = 38)	On-call duty, nighttime (*n* = 106)	
Interventionalist’s experience				
< 1 year	36 (35%)	11 (29%)	28 (26%)	*p* = 0.37
> 1 year	66 (65%)	27 (71%)	78 (73%)	
i.v. lysis	47 (46%)	18 (47%)	64 (60%)	*p* = 0.11
Number of maneuvers	1.78 ± 1.42	1.76 ± 1.26	2.26 ± 2.02	*p* = 0.30
MT technique				
Aspiration	14 (14%)	5 (13%)	20 (19%)	*p* = 0.53
Asp. + stent retriever	88 (86%)	33 (86%)	86 (81%)	
Fluoroscopy time (min)	14 [7–23.25]	15.5 [7.75–28.5]	15 [7–23]	*p* = 0.64
DAP (Gy*cm^2^)	49.11 [26.3–80.63]	49.20 [29.98–91.31]	50.34 [31.19–86.48]	*p* = 0.89
Contrast medium (ml)	120.7 ± 47.70	111.1 ± 32.37	118.7 ± 37.92	*p* = 0.47
Time interval (min)				
onset- groin puncture	180 [125.5–237]	190 [148.5–285]	185.5 [138.8–242.3]	*p* = 0.27
admission- groin puncture	74 [33–116]	87.50 [44.25–123.3]	86 [29.5–112.5]	*p* = 0.64
admission- recanalization	132 [80.5–170.8]	137 [101.8–177]	128 [81.5–165]	*p* = 0.77
groin puncture- recanalization	40 [21–71]	36 [24.75–76.50]	40 [23–70]	*p* = 0.93

*Asp.*, aspiration; *DAP*, dose area product; *MT*, mechanical thrombectomy

In comparison to the data provided by the German Federal Office for Radiation Protection, these were in the range of the 25th percentile of the average radiation exposure during aspiration thrombectomy (25th percentile 51 Gy*cm^2^), while the recommended threshold or DRL was defined as the 75th percentile (180 Gy*cm^2^) [7].

A more detailed analysis showed that the corresponding DRL was exceeded only outside of the regular operating hours—twice during nighttime on-call duty and once during daytime on-call duty (standard working hours 0% (0/102), daytime/nighttime on-call duty 2.1% (3/144), *p* = 0.27). Exceeding the 50th percentile (91 Gy*cm^2^) was also more frequent during daytime and nighttime on-call duty (standard working hours 4.9% (5/102), daytime/nighttime on-call duty 12.5% (18/144), *p* = 0.047). No further significant differences were documented for the other parameters such as fluoroscopy time, procedure time (groin puncture to recanalisation) or interventionalist’s experience.

### Outcome

With regard to the outcome parameters (shown in Table 3) there was a significant higher in-hospital mortality during daytime and nighttime on-call duty when compared to regular hours (standard operational hours 7% (7/102), daytime on-call duty 16% (6/38), nighttime on-call duty 20% (21/106), *p* = 0.02). Control imaging showed no significant differences in postinterventional sICH or SAH (*p* > 0.05 each).

**Table 3:** Outcome

	Standard working hours (*n* = 102)	On-call duty, daytime (*n* = 38)	On-call duty, nighttime (*n* = 106)	
Final TICI				
1st pass TICI3	39 (38%)	13 (34%)	36 (34%)	*p* = 0.79
TICI ≥ 2b	97 (94%)	36 (95%)	96 (91%)	*p* = 0.40
TICI 3	55 (54%)	19 (50%)	63 (59%)	*p* = 0.54
sICH	7 (7%)	3 (8%)	7 (6%)	*p* = 0.96
SAH	13 (12%)	3 (7%)	6 (5%)	*p* = 0.20
In-hospital mortality	7 (7%)	6 (16%)	21 (20%)	*p* = 0.02*
NIHSS discharge	7 (3–16)	10 (2–16)	11 (3–23.5)	*p* = 0.45
mRS 0–2 discharge	35 (34%)	9 (24%)	30 (28%)	*p* = 0.41
mRS 0–2 90 days	35 (34%)	9 (24%)	29 (27%)	*p* = 0.37
ASPECTS post	7 (6–9)	7 (5.25-8.75)	7 (5–9)	*p* = 0.55

*ASPECTS*, Alberta Stroke Program Early CT Score; *mRS*, modified Rankin scale score; *NIHSS*, National Institutes of Health Stroke Scale Score; *TICI*, thrombolysis in cerebral infarction; *SAH*, subarachnoid haemorrhage; *sICH*, symptomatic intracerebral haemorrhage

*Statistical significance, *p* < 0.05

A subsequent analysis of the causes of death is shown in Table 4. The most frequent cause was the wish for therapy limitation, followed by sepsis, respiratory insufficiency and ICH. In the statistical analysis, no cause of death was significantly more frequent than the others within each of the three collectives. In the comparison between the different collectives, the distribution of causes of death in the nighttime on-call duty collective was statistically different (*p* < 0.05) than in the standard working hours and in the daytime on-call duty collective, with fewer cases of therapy limitations but more cases of all other categories.

**Table 4:** Causes of death

Causes of death (total *n* = 34)	Standard working hours (*n* = 7)	On-call duty, daytime (*n* = 6)	On-call duty, nighttime (*n* = 21)
ICH	1 (14.29%)	1 (16.67%)	3 (14.29%)
Sepsis	0	1 (16.67%)	7 (33.33%)
Respiratory insufficiency	2 (28.57%)	1 (16.67%)	4 (19.05%)
Therapy limitation	4 (57.14%)	2 (33.33%)	3 (14.29%)
Other	0	0	3 (14.29%)
Unknown	0	1 (16.67%)	1 (5.76%)

*ICH*, intracranial haemorrhage

There was no significant difference between the scores recorded at hospital discharge and the mRS score recorded after 90 days. Regarding the reperfusion, a favourable result (TICI ≥ 2b) was achieved in the majority of cases (> 90%), without significant differences between the collectives (*p* > 0.05, each).

### Multivariate analysis

Multiple linear regression analyses for the parameters DAP, fluoroscopy time and procedural time as well as a multiple logistic regression analysis for the parameter mortality were performed and summarised in Table 5. From the factors examined, only gender could be identified as a significant confounder on the radiation exposure in our collective (*p* = 0.041). Male gender could be identified as a risk factor for higher radiation exposure in our collective (DAP [Gy*cm^2^]: male 57.73 [36.27–89.63], female 42.18 [24.38–72.94]).

**Table 5:** Multivariate analysis

	DAP	Fluoroscopy time	Procedural time	Mortality
Gender	*p* = 0.041*	n. s.	n. s.	n. s.
Age	n. s.	n. s.	n. s.	*p* = 0.017*
Interventionalist’s experience	n. s.	*p* = 0.0002***	*p* < 0.0001****	n. s.
ASPECTS (baseline)	n. s.	n. s.	n. s.	*p* = 0.0002***
aHTN	n. s.	*p* = 0.045*	*p* = 0.011*	n. s.
AF	n. s.	n. s.	n. s.	n. s.
DM	n. s.	*p* = 0.019*	n. s.	n. s.

*AF*, atrial fibrillation; *aHTN*, arterial hypertension; *ASPECTS*, Alberta Stroke Program Early CT Score; *DAP*, dose area product; *DM*, diabetes mellitus

*Statistical significance, *p* < 0.05

**p < 0.01

***p < 0.001

****p < 0.0001

With regard to fluoroscopy time, the experience of the interventionalist and the cardiovascular risk factors arterial hypertension and diabetes mellitus were significant confounders. With the exception of diabetes mellitus, these were the same factors that showed a significant influence on the total procedural time. With regard to mortality, age and the baseline ASPECTS were significant confounders.

## Discussion

In this study, we compared baseline characteristics, procedural specifics including dosimetrics and outcome data in patients who underwent MT due to LVO in anterior circulation within and outside regular working hours. Similar to other authors, we found an increased in-hospital mortality in patients treated during daytime and nighttime on-call duty [3]. However, our data suggests that this worse patient outcome cannot be entirely explained by the technical performance of MT itself. With regard to the DAP, no significant differences regarding an exceeding of the DRL or a significant difference regarding the DAP median values could be shown with median DAP values in the range of the 25th percentile of the average DAP of the German Federal Office for Radiation Protection. The multivariate analysis of further possible confounders on radiation exposure showed a significant influence of the factor gender on radiation exposure, revealing male gender as a risk factor for higher radiation exposure. However, since there were no significant differences in the gender distribution within the collectives we selected, no impact on the results we obtained is to be expected. This finding has already been described by Farah et al [9], who attributed it to a generally heavier weight of men compared to women. This correlation can be explained, for example, by an automated adjustment of the irradiation parameters of the angiography system depending on the patient’s weight to improve the image quality. Also, differences due to vascular anatomy and differences in degenerative and arteriosclerotic changes of vessels related to the patient’s gender seem possible, e.g. due to a more difficult access route and the resulting need for more images.

The surrogate parameters selected in addition to DAP—fluoroscopy time and procedural time—showed no significant differences between groups and thus did not indicate a more difficult performance during daytime and nighttime on-call duty. Compared to other studies, our study exhibited comparatively low fluoroscopy and overall procedure time periods [8, 9]. With regard to multivariate analysis, the experience of the interventionalist had a significant influence on fluoroscopy and procedural time, however, not on radiation exposure and mortality. Since the proportion of less experienced interventionalists did not differ significantly between the groups, no influence on the overall result is to be expected. The presence of arterial hypertension or diabetes also showed a significant influence on fluoroscopy time and in the case of arterial hypertension also on the overall procedural time. This could, for example, be explained by increased atherosclerotic changes in the vascular access route of patients suffering from these conditions and correspondingly more difficulties in reaching the intervention site. However, again, there were no significant differences between the collectives regarding these conditions, so that an influence on the overall result seems unlikely. As a further technical aspect, the number of thrombectomy manoeuvres was also examined and showed a tendency towards an increased number of thrombectomy manoeuvres at night, but without reaching the significance level.

Compared to other studies, no significantly prolonged admission-to-groin puncture interval could be determined [2], although a tendency towards longer admission-to-groin puncture intervals during daytime and nighttime on-call duty is shown with a difference of about 10 min (median admission-to-groin puncture interval in minutes: standard operational hours 74 [33-116], daytime on-call duty 87.5 [44.25-123.3], nighttime on-call duty 86 [29.5-112.5], *p* > 0.05). These differences are certainly at least in part dependent on individual hospital structures and organisational factors, so that generalizability of these findings is limited. Eventually, each neurovascular centre should review its performance as in-house quality control and restructure if necessary.

Our multivariate analysis showed a significant influence of the factors age and baseline ASPECTS on mortality, which is obvious and corresponds to the results of previous studies [17]. However, there were no significant differences between the collectives regarding these aspects either. Also, the complications we examined (SAH, sICH) did not significantly differ between groups and thus not explain the differences in mortality shown.

Since there were no significant differences between the detected confounders of mortality, age and baseline ASPECTS, other reasons must be held responsible for the observed differences in mortality. A further analysis of the causes of death revealed the wish for treatment limitation as the main cause of death (more frequent in the standard working hours collective), followed by sepsis, respiratory failure and ICH (all more frequent in the night on-call collective). Explanations as to why these conditions occur more frequently in the nightly treated collective remain speculative.

The subject examined in our study has already been partially addressed in previous publications with varying results. While Nikoubashman et al [18] and Wedell et al [19] could not demonstrate any effect of the time of admission on the outcome or radiological features, Ogbu et al [3] and Roberts et al [20] showed increased mortality at the weekend and especially at night. Saad et al [21] were able to demonstrate increased mortality at weekends only in non-teaching hospitals, which was attributed to the fact that non-teaching hospitals are less often certified stroke centres and have less specialised personnel, such as vascular neurologists, leading to less guideline adherent behaviour, especially at night and on weekends. Wedell et al showed no effect on outcome, but differences in the patient populations admitted at different times as well as admission-to-groin time.

In relation to our study, this means that various causes have to be discussed in relation to the increased mortality. These include a reduction in the quality of inpatient care due to reduced and less experienced staff during night hours, which could lead to delayed detection of complications and consequently delayed start of therapy. Differences in the enrolled patient populations in terms of the clinical status prior to ictus (e.g. with pre-morbid mRS as a surrogate) including previous stroke events may also have been a factor. However, a detailed analysis of all possible causes for the differences in mortality found was, based on the available data, not possible in this study and should be the subject of further studies.

The basic limitation of this study is the single-centre retrospective design. Furthermore, only DAP and fluoroscopy time were determined to define radiation exposure; there was no consideration of other dosimetrics, such as peak skin dose and air kerma product.

## Conclusion

Our data indicate a “weekend effect” in terms of patient outcome, with increased in-hospital mortality in patients treated with MT outside the standard operational hours. Since no significant differences in procedural specificities were found, this effect cannot necessarily be explained by inferior quality of the performed MT. In particular, the surrogate parameters radiation exposure, fluoroscopy time and procedural time were independent from the time of treatment (standard operating hours vs. daytime/nighttime on-call duty). It remains debatable whether other factors outside the responsibility of the treating neurointerventionalist might contribute to the different patient outcome. Further multi-centre studies with larger patient numbers are needed.

## Abbreviations

aHTN: Arterial hypertension
AIS: Acute ischemic stroke
ASPECTS: Alberta stroke program early CT score
CTA: Computed tomography angiography
DAP: Dose area product
DRL: Diagnostic reference level
DSA: Digital subtraction angiography
DWI: Diffusion weight imaging
F: French
ICH: Intracranial haemorrhage
IQR: Interquartile range
LVO: Large vessel occlusion
MCA: Medial cerebral artery
MRI: Magnetic resonance imaging
mRS: Modified Rankin scale score
MT: Mechanical thrombectomy
NCCT: Native cranial computed tomography
NIHSS: National Institutes of Health Stroke Scale Score
sICH: Symptomatic intracerebral haemorrhage
TICI: Thrombolysis in cerebral infarction
